# Musical harmony is processed during sleep: a proof-of-concept study

**DOI:** 10.1093/sleepadvances/zpaf085

**Published:** 2025-11-25

**Authors:** Anna Zoé Wick, Björn Rasch

**Affiliations:** Department of Psychology, University of Fribourg, Fribourg, Switzerland; Department of Psychology, University of Fribourg, Fribourg, Switzerland

**Keywords:** sleep, musical harmony, EEG analysis, event-related potentials

## Abstract

Musical chords represent the most basic musical elements, conveying emotional information. During wakefulness, different musical chord categories elicit distinct neuronal correlates and emotions, with major chords typically inducing more positive and minor chords negative ones. However, it remains unclear whether the brain continues to process musical chords differently when presented during sleep. To address this question, we conducted a proof-of-concept study and presented musical major, minor, and dissonant chords to 47 healthy participants during nocturnal non-rapid eye movement sleep. Prior to sleep, participants rated the chords in valence and arousal. Sleep was recorded using polysomnography. Our analysis of event-related responses during sleep revealed significant differences between the three musical chord categories. Major chords induced the strongest negative amplitude approximately 800 ms after chord onset, indicated as peak-to-peak (PTP) amplitude from the earlier positive peak. Minor chords showed intermediate PTP amplitudes, while dissonant chords elicited the lowest PTP amplitudes. In the time-frequency domain, these differences were also apparent, including differences in slow-wave, theta, alpha, and sleep spindle bands across chord categories. Notably, experience in playing a musical instrument induced stronger differentiation of musical harmony during sleep compared with participants who never played a musical instrument. In conclusion, our findings suggest that the different processing of single musical chords persists during sleep, influenced by harmonic features and musical expertise in the context of Western musical conventions. Future research should explore whether longer and more complex harmonic features (e.g. chord sequences or musical pieces) are differentially processed by the sleeping brain.

**Statement of significance**

Whether the sleeping brain can process musical harmony remains an open question. In this study, we investigated the perception of musical harmony during sleep. Our results demonstrate that the sleeping brain is capable of differentiating between musical chords, and that this sensitivity is modulated by the level of musical expertise. Given the strong link between musical harmony and emotional processing, these findings have important implications for the use of music to influence emotions during sleep and dreaming and may even contribute to novel approaches for improving sleep quality in clinical settings.

## Introduction

A musical chord can be considered as the smallest musical element that conveys emotional information [1, 2]. It consists of the simultaneous play of more than two tones and encompasses both consonant and dissonant intervals [3, 4]. Consonant intervals that form major and minor chords sound stable and complete, whereas dissonant intervals are restless and seek resolution in consonant intervals [3, 4].

Consonant and dissonant chords are fundamental elements to Western music [5], eliciting distinct emotional responses in listeners [1, 5]. Minor chords are less harmonious than major chords and research has shown that minor chords are generally perceived more negatively than major chords, which tend to connote more positive emotions [4, 6, 7]. Dissonant chords have been found to induce unpleasant feelings [8–10]. The association between major and minor tonalities and their respective positive and negative emotional valences has been found to be psychologically robust [11, 12].

This emotional perception appears to be, at least partially, independent of formal musical training [8, 9, 13, 14], as it may be rooted in low-level sensory processing [15]. However, the affective response to musical stimuli seems to involve a complex interplay of factors, potentially including learned associations between musical elements and emotional states, prior musical experience, and psychoacoustic properties of the auditory stimulus [16]. For example, musical expertise seems to enhance the sensitivity to harmonic structures and the consonance-dissonance perception [17].

Several neuroimaging studies have investigated the perception of musical chords during wakefulness [9, 18–20]. All these studies consistently show that consonant major, minor, and dissonant chords can elicit distinct neural correlates and activate different brain areas.

For example, Maslennikova et al. [21] found higher event related potentials (ERPs) elicited by consonant than dissonant chords in early ERP components (P100 and N200). In addition, differences in the mismatch activity were observed between harmonic and dissonant chords [22, 23].

When comparing musicians and non-musicians, ERP amplitudes of early components of dissonant and consonant chords were significantly smaller in non-musicians [8]. To our knowledge, the brain response to isolated musical chords during sleep has not yet been systematically examined.

Generally, it is now widely accepted that the sleeping brain is capable of processing and distinguishing auditory stimuli. For example, sleep studies have confirmed that a person’s own name, but also affective qualities, such as the emotional prosody or familiarity of voices or the “roughness” of shouts, can be distinguished during sleep [24–29].

In sleep and memory research, the capability of the sleeping brain to processes auditory stimuli is used to reactivate and strengthen associated memories in targeted memory reactivation (TMR) studies [30, 31]. TMR is a prominent technique to investigate the process of memory consolidation by presenting reminder cues during sleep, such as odors, tones, clicks, or words that were linked to learned material before sleep [30]. TMR enables the targeted modulation of memory consolidation by reactivating and reinforcing particular memory traces [30, 32]. The effects of these externally triggered reactivations on memory performance can be assessed immediately after sleep [31, 33]. TMR generally enhances memory performance following sleep and has been effectively employed for declarative, procedural, and emotional memory [30, 34].

While some TMR studies have used musical stimuli as reactivation cues during sleep successfully [35–37], it has not yet been examined to what extent music itself can be processed during sleep. One recent study started to fill this gap and investigated rhythm perception during sleep and found that it is only partially possible during sleep [38]. Given that music evokes strong emotions and musical chords represent the smallest musical elements conveying emotional information [1], using musical elements similar to TMR-cues could provide a novel way to examine whether such cues can reactivate emotional memories or emotions and potentially modulate even sleep quality during sleep.

Therefore, we conducted a first proof-of-concept study to explore the fundamental question of whether musical chords as the smallest musical elements are even perceived during sleep. In particular, we examined whether different types of chords elicit distinguishable ERPs in the sleeping brain.

We conducted an experiment including 47 young and healthy participants who spent one experimental night in the sleep laboratory. We presented different isolated chord types with no harmonic context (major chords, minor chords, and dissonant triads including whole tone and half tone intervals) in a randomized order during non-rapid eye movement (NREM) sleep stages N2 and SWS. We hypothesized that different chord types elicit distinct ERPs and oscillatory activity in both N2 and SWS sleep stages.

## Materials and Methods

### Participants

Our final sample consists of 47 healthy young participants (ages 19 to 25; *M* = 21.26; *SD* = 1.57; 35 females). The final sample encompasses data from our pilot study with 12 young and healthy participants (8 females) ages 21 to 25 (*M* = 22.00; *SD* = 1.28). After our pilot study, we preregistered our study (osf.io/yq7zw) and conducted our main study, including 35 young and healthy participants (27 females) ages 19 to 25 years (*M* = 21.00; *SD* = 1.59). As the procedure of the pilot and main study are identical and the results turned out to be highly comparable, we combined both data sets to improve statistical power.

All subjects were German or French speakers, were free of any physical and mental disease, and reported a healthy wake–sleep cycle. We recruited subjects via a newsletter sent to psychology students and advertisements on several internet platforms. All subjects were instructed to maintain their regular sleep–wake cycle throughout the entire experiment. Two days before each session, subjects received a reminder e-mail containing the instructions to refrain from consuming caffeine and alcohol for two days prior to the experiment. Additionally, we asked subjects to get up no later than 07:00 am on the day of the experiment.

The studies were approved by the local ethics committee, and all subjects gave written informed consent at the beginning of the adaptation night. The study was conducted in accordance with the Declaration of Helsinki. Subjects received compensation of 80 CHF for their participation. In case of early abandonment, we paid subjects proportionally.

### Design and procedure

The study consisted of an adaptation and one experimental night in the sleep laboratory at the University of Fribourg. The nights were separated by a minimum of three and a maximum of 28 nights. During the experimental night, subjects arrived at the sleep laboratory at 08:15 pm, filled out various questionnaires, and prepared for the night. Next, the experimenter attached the electrodes for the polysomnographic (PSG) recordings, and the subjects listened to the musical chords while playing a simple puzzle game (Block Game) for 45 min. Before and after this game, participants rated the different chords by valence and arousal. At approximately 11:00 pm, the subjects went to bed, and the lights were turned off for eight hours. After sleep onset and entering NREM sleep, the experimenter manually started to play the musical chords using E-Prime 2.0.10. After approximately 3.5 h of sleep, the experimenter stopped the manual reactivation program and initiated an automatic presentation of the chords for the remaining sleeping time. In the morning, the experimenter turned on the light and woke up the subject. Participants immediately filled out questionnaires, listened to the musical chords while playing the puzzle game, and rated the different chords by valence and arousal before and after this morning’s task. Afterwards, the experimenter detached the electrodes, and the subjects left the laboratory between 09:00 and 09:30 am.

### Questionnaires

Prior to the experimental session, participants completed several questionnaires to obtain information about demographics, handedness, drug consumption, and personality. We used the Pittsburgh Sleep Quality Index [39] to gain information about the subjective sleep quality and sleep habits during the last four weeks. We also evaluated the musical expertise of the participants using the MUSE questionnaire [40]. The questionnaire explores a variety of aspects of musical engagement, including the question of whether participants have ever played or currently play a musical instrument, the amount of time spent listening to music during the week, and the role that music plays in their cognitive and emotional regulation. Finally, to determine the current mood of the subjects, we used the standardized Multidimensional Mood Questionnaire (MDBF) [41], in which subjects rated their present mood using a Likert scale on multiple items.

In the following morning, we operationalized the subjective sleep quality from the previous night using the SF-A/R questionnaire [42], and once more, we collected data on the subjects’ moods using the MDBF [41].

Moreover, to measure subjects’ affective reaction to the stimuli, they rated the musical chords, and we assessed the dimensions “Valence” and “Arousal” with the Self-Assessment Manikin questionnaire [43] which employed a nine-point scale before and after sleep.

### Stimuli

**Figure 1:**
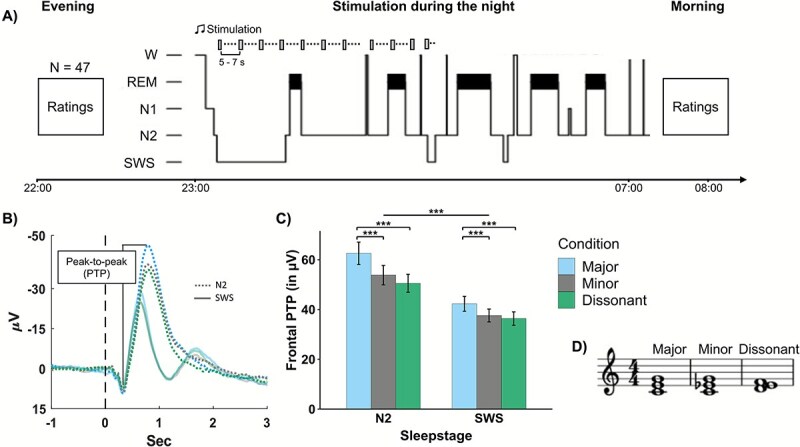
Experimental procedure, behavioral results and notation of chord stimuli. (A) Experimental procedure. After preparation in the evening, 47 participants rated the different chords according to their valence and arousal. Then, the participants went to bed and slept for 8 h. During sleep, we presented major, minor, and dissonant chords with an interstimulus interval of 5 to 7 s. In the morning, participants listened to the chords and rated them again. (B) The averaged frontal ERP response (in μV) recorded with F3 and F4 after playing major, minor, and dissonant chords during sleep. Chords were played at time 0. Frontal PTP amplitudes were calculated by subtracting the positive peak from the negative peak. (c) Means ± SEM as a bar graph of PTP amplitudes (in μV) separated by musical chord condition and sleep stage. Major vs. minor chords and major vs. dissonant chords differed significantly from each other in both sleep stages. In addition, both sleep stages N2 and SWS differed significantly from each other indicated by ^***^: *p* < .001. (D) notation of major (C-E-G), minor (C-E*^b^*-G), and dissonant (D-E-F) chords.

We used the programs GarageBand 10.3.2 (Apple Inc., 2019) and Adobe Audition 22.6 (Adobe Inc., 2022) to create the musical stimuli for the study. The grand piano chords were each 2 s long and were triads following major, minor, and western dissonant characteristics. We composed two basic consonant chords: the major chord was constructed with a major third below and a minor third above, and a minor chord with a minor third and a major third above. We also created a dissonant chord, which consisted of a triad that included a whole tone (major second) below and a half tone (minor second) interval above. These intervals are considered dissonant [4, 44]. To add more variability, we transposed the pitch of the individual chords by four and six semitones higher and lower. The notation of the basic chords is presented in Figure 1.

### Task before and after sleep

We used E-Prime software 2.0.10 (Psychology Software Tools Inc., Pittsburgh) to program the randomized musical chord presentation. The task lasted 45 min and consisted of two 20 min parts, which were separated by a 5 min break. To add more variability for the participants, we implemented not only single major, minor, and dissonant chords but also major, minor, and dissonant chord sequences, which, however, were not further used or analyzed.

In both parts, chords are presented acoustically via loudspeaker (range of chord duration: 2–6 s) repeatedly and in a randomized order (notation of chords see Figure 1, B). An interval of 1000–3000 ms between each musical stimulus reduced the presentation speed of chords. During the two parts, subjects were instructed to move as little as possible to play a simple puzzle game at the computer while the chords were played in the background. After 20 min and a presentation of 240 stimuli, a 5 min break followed. Then, the second part followed, which was identical to the first part.

### Reactivation of musical chords during the night

Again, we used E-Prime 2.0.10 (Psychology Software Tools Inc., Pittsburgh), to acoustically play the musical chords at a sound pressure level of 35 dB during sleep via loudspeakers and presented them in a random order. Chords were separated by an interstimulus interval of 5000–7000 ms to avoid a regular rhythm of chord presentation.

As soon as signs of stable sleep appeared (as given by the American Association of Sleep Medicine [AASM] [45]), the experimenter started the presentation of chords manually and paused immediately when signs of arousals or awakenings were detectable. After approximately 3.5 h, the experimenter terminated the manual reactivation procedure. On average, we presented 363.5 **±** 92.6 repetitions of major, minor, and dissonant chords during N2 and SWS.

### PSG recordings

We collected electrophysiological data during sleep using different PSG components: the electroencephalogram (EEG) consisted of twelve single gold-cup electrodes, which we positioned in accordance with the international 10–20 system (C3, C4, Cz, F3, F4, Fpz, P3, P4, M1, M2, O1, O2) [46]. Moreover, we attached the electromyogram (EMG), the electrooculogram (EOG) and the electrocardiogram (ECG) according to the international recommendation AASM [45]. We derived the EEG referentially, whereas the EMG, EOG, and ECG were generated bipolarly. However, we maintained all impedances below 5 kΩ and set the sampling rate to 500 Hz, along with the low- and high-frequency recording filters of each component set to the suggested level of the AASM (EEG: 0.3/35 Hz; EOG: 0.3/35 Hz; EMG: 10/100 Hz; ECG: 0.3/70 Hz [45]).

### Sleep scoring

The electrophysiological data was first re-referenced to the contralateral mastoid and filtered as recommended by the AASM [45] using the software BrainVisionAnalyzer 2.2 (Brain-Products Inc., GmbH, Gilching, Germany). Then, we segmented the 8 h of bedtime (from “Lights off” to “Lights on”) into 30-s epochs and two independent scorers visually classified the epochs with the different stages “Wake,” “REM,” “N1,” “N2,” or “SWS.” In case of disagreements, we consulted a third expert sleep scorer.

### Event related analyses

To gain further insights into the oscillatory activity of the manual chord presentation during N2 and SWS, we inspected event-related potentials (ERPs) and performed time–frequency analyses (TFA). We used the BrainVisionAnalyzer 2.2 (Brain Products Inc., GmbH, Gilching, Germany) to first preprocess the EEG data by removing artifacts from the data. At the beginning of this process, we imported sleep scorings to serve as segmentation markers. Based on both NREM sleep stages, N2 sleep and SWS, chords were separated into equally sized segments with borders set at 3 s before and 5 s after a musical chord. Then, the following steps were performed following the procedure described by Ackermann et al. [47] A high-pass filter was set to 0.1 Hz, a low-pass filter to 40 Hz, and a notch filter to 50 Hz. Finally, the data were re-referenced to the averaged value of both mastoids. To ensure that we only analyze artifact-free sections, an automatic artifact rejection excluded segments, if the maximal difference in EMG activity was above 150 μV and the maximal difference in each EEG channel was above 500 μV.

For the ERP analyses, the baseline was normalized using a period of –1 to 0 s before stimulus onset. Afterwards, for all subjects and for each musical chord category, data was averaged over both sleep stages. The maximal peak of the event-related response was then exported using automatic peak detection and further analyzed in R Studio software 4.3.1 (RStudio Team, Boston).

For the TFA, artifact-free segments were exported and further analyzed with the Fieldtrip toolbox [48]. The TFA procedure was adapted from Beck et al. [49]. Similarly, the baseline was normalized with a period of –1 to 0 s before stimulus onset. In the next step, we averaged the data per subject and per chord category to compare oscillatory power across different frequencies (1–20 Hz) within a time window of 0 to 4.5 s after chord onset. This was done for the averaged power of both frontal channels (F3, F4). Results were compared using cluster-based permutation tests for dependent samples as implemented in the FieldTrip toolbox [48]. For the cluster-level statistics, we used the maximum sum of t-values within each cluster. The cluster-level statistic was calculated for each of 1000 randomly drawn data partitions, which exceed the actually observed test statistics, resulting in a Monte Carlo *p*-value, controlling for multiple comparisons. The alpha level was set to .05 and corrected for two-sided testing.

We compared major, minor, and dissonant chords played during N2 and during SWS. We did this for all chords, separately for each sleep stage.

### Statistical analyses

We carried out all analyses using the R Studio software 4.3.1. In order to investigate the differences in ERPs of major, minor, and dissonant chords in NREM sleep stages N2 and SWS, as well as within the subjective valence and arousal ratings, we ran several sets of mixed-design analyses of variances (ANOVAs). When Mauchly’s tests revealed a violation of sphericity, the correction proposed by Greenhouse–Geisser was used. Effect patterns were further explored by post hoc *t*-tests. For all analyses, the significance level was set to *p* ≤ .05. In the case of multiple post hoc testing, we used the Bonferroni correction to minimize potential errors. In addition, we calculated differences between two chord categories (Major-Minor, Major-Dissonant, and Minor-Dissonant) for several parameters of interest and used these differences to explore correlations and modulations.

## Results

To test our hypotheses, 47 participants spent one experimental night in the sleep laboratory at the University of Fribourg (see the Materials and Methods section for more details; see Table 1 for an overview of the sleep architecture). During the nocturnal sleep, single major, minor, and dissonant-sounding chords in different pitches were presented at a low volume during stable NREM sleep stages N2 and slow-wave sleep (SWS). We set a random intertrial interval between 5 and 7 s to avoid a regular rhythm of stimuli presentation, attenuating the response [50]. Sleep was recorded using polysomnography involving 12 EEG electrodes.

**Table 1:** Overview of times spent in the different sleep stages during the experimental night

**Sleep parameter**	**Mean**	**±**	**SEM**
TST (min)	437.54	±	4.47
WASO (%)	5.09	±	0.50
N1 (%)	3.79	±	0.27
N2 (%)	48.45	±	1.18
SWS (%)	21.51	±	1.09
REM (%)	21.17	±	0.73
SWS latency (min)	25.04	±	3.92
REM latency (min)	101.07	±	7.50

Times in the different sleep stages. SOL: Sleep onset latency; WASO: wake after sleep onset; N1, N2: NonREM sleep stages N1 and N2; SWS: slow wave sleep; REM: rapid-eye movement sleep; TST: Total sleep time. Numbers are means ± SEM (in min or %).

### Subjective rating

Before and after sleep, subjects rated major chords highest in valence (6.56 ± 0.17) and lowest in arousal (3.47 ± 0.26). Minor chords received intermediate ratings in valence (5.28 ± 0.21) and arousal (4.17 ± 0.24), while dissonant chords were rated lowest in valence (3.44 ± 0.17) and highest in arousal (4.96 ± 0.23). The valence and arousal ratings of all three chord types differed significantly (valence: *F*(1.69, 77.59) = 99.65, *p* < .001, *η_G_*^2^ = 0.52), arousal: *F*(1.54, 70.63) = 35.04, *p* < .001, *η_G_*^2^ = 0.12, see Table 2 for descriptives and ANOVA statistics).

**Table 2:** Subjective Rating and ERP features of major, minor, and dissonant chords

										**ANOVA**		
**Subj. Rating**	**Major**			**Minor**			**Dissonant**			**Chord type**	**Sleep stage**	**Interaction**
	Mean	±	SEM	Mean	±	SEM	Mean	±	SEM	***F***	***F***	***F***
Valence	6.56	±	0.17	5.28	±	0.21	3.44	±	0.17	99.41^***^	n.e.	n.e.
Arousal	3.47	±	0.26	4.17	±	0.24	4.96	±	0.23	34.25^***^	n.e.	n.e.
**ERP**												
PTP (μV)												
N2	**62.58** ^**a,b**^	±	4.5	53.81	±	3.91	50.54	±	3.63	26.73^***^	33.1^***^	5.28^*^
SWS	**42.3** ^**a,b**^	±	3.00	37.61	±	2.58	36.36	±	2.69			
PP (μV)												
N2	11.09	±	0.87	9.95	±	0.97	8.85	±	0.73	5.66^**^	0.09	0.01
SWS	10.78	±	0.78	9.8	±	0.8	8.61	±	0.87			
Latency PP (ms)												
N2	312.6	±	5.37	322.38	±	7.21	327.06	±	7.6	0.49	3.23^#^	**5.19**
SWS	311.53	±	8.12	307.74	±	10.46	290.81	±	9.31			
NP (μV)												
N2	**−51.49** ^**a,b**^	±	4.1	−43.87	±	3.43	−41.7	±	3.5	16.39^***^	40.43^***^	5.38^**^
SWS	−31.52	±	2.85	−27.8	±	2.34	−27.75	±	2.54			
Latency NP (ms)												
N2	818.34	±	16.43	791.79	±	19.9	801.36	±	17.87	1.8	156.04^***^	0.14
SWS	647.91	±	13.42	634.09	±	13.94	643.19	±	10.81			

Data are means ± SEM. Subjectively perceived valence and arousal of major, minor, and dissonant chords are shown, as well as ANOVA test statistics including the factors Condition (Major, Minor, and Dissonant), Sleep stage (N2, SWS) as well as a possible interaction of these two factors (*F*; significant *F*-values in bold). Additionally, peaks (in μV) and latencies (in ms) of the ERPs elicited during N2 sleep stage or slow-wave sleep (SWS) are presented. PTP: Peak-to-peak refers to the difference between the positive minus the negative peak; PP: positive peak; Latency PP: latency of the positive peak; NP: negative peak; Latency NP: Latency of the negative peak. Significant differences between conditions are in bold and are indicated by a = significant difference between this category and minor chords and b = significant difference between this category and dissonant chords. Numbers are means ± SEM (in ms or μV). Significant effects are indicated in numbers and by ^*^: *p* < .05; ^**^: *p* < .01; ^***^: *p* < .001. Non-existent effects are indicated by n.e.

### Event related responses to musical chord presentation during sleep

Musical chord processing during NREM sleep elicited a very stable event-related response (ERP), which was generally higher in N2 sleep compared with SWS (see Figure 1, D). Importantly, the ERP elicited by musical chords during sleep differed significantly between chord types, confirming our hypothesis: During N2 the PTP difference was at 55.64 ± 4.03 μV and lower during SWS at 38.76 ± 2.76 μV. Moreover, we observed the largest peak-to-peak (PTP) differences for major chords (N2: 65.58 ± 4.5 μV; SWS: 42.3 ± 3.00 μV), with lower PTP differences for minor (N2: 53.81 ± 3.91 μV; SWS: 37.61 ± 2.58) and dissonant chords (N2: 50.54 ± 3.63 μV; SWS: 36.36 ± 2.69 μV), similarly in both sleep stages N2 and SWS (see Figure 1, C and Table 1, for sleep stage-specific results). The PTP was calculated between the first positive peak and the first negative peak (NP) of the ERP. The main effect of chord type was highly significant (*F*(2,92) = 26.73, *p* < .001, *η_G_*^2^ = 0.03). Post hoc pairwise *t*-tests remained significant after Bonferroni corrections (*p*_corrected*​*_ = 0.00833) for the PTP comparison for major vs. minor chords as well as major vs. dissonant chords (all *p* < .001, see Table 2). No significant difference in PTP was observed between minor and dissonant chords.

When comparing NREM sleep stages N2 and SWS, we observed a highly significant main effect of sleep stage confirming that ERP responses to chords were generally higher in N2 compared with SWS (*F*(1,46) = 33.10, *p* < .001, *η_G_*^2^ = 0.11). Additionally, we detected a significant interaction between Chord type and Sleep stage (*F*(1.61, 74.03) = 5.28, *p* = .011, *η_G_*^2^ = 0.01). The interaction is driven by generally larger differences in PTP amplitudes between the different chord types in N2 compared with SWS (e.g. the difference in PTP amplitudes between minor and dissonant chords reached a statistical trend in N2 (*p* = .091) but was not significant in SWS (*p* = .294).

The latencies of the positive and negative peaks did not differ significantly between the musical chord types (main effect of chord type *p* > .171, see Table 2). Generally, for all chord types, the negative peak occurred later in N2 sleep (803.83 ± 18.14 ms) compared with SWS (641.73 ± 12.79 ms), corresponding to the higher amplitude elicited in N2 sleep (*F*(1,46) = 156.04, *p* < .001, *η_G_*^2^ = 0.37). The latency of the positive peak was also descriptively later in N2 sleep vs. SWS (320.68 ± 6.79 ms vs. 303.36 ± 9.34 ms), but did not significantly differ between sleep stages (*p* > .05). Additionally, we observed a significant interaction for the latency of the positive peak (*F*(1,46) = 5.19, *p* = .007, *η_G_*^2^ = 0.02): While the latency of the positive peak was shortest for major chords in N2 sleep, it was shortest for dissonant chords in SWS (see Table 2). However, subsequent post hoc *t*-tests did not remain significant after Bonferroni correction (all *p* > .01; see Table 2).

In conclusion, the PTP amplitudes elicited by major, minor, and dissonant chords during sleep differed significantly, both during N2 sleep and SWS. These findings suggest that our brain is capable of differentially processing musical chords according to their harmonic properties during NREM sleep.

### Oscillatory correlates of musical chord presentation during sleep

**Figure 2:**
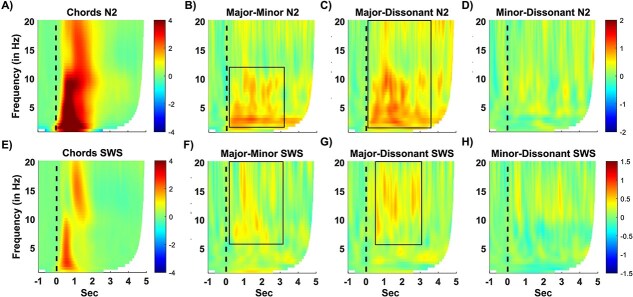
Oscillatory responses to chords presented during N2 and SWS in the time-frequency spectrum recorded at electrodes F3 and F4. (A) Oscillatory power changes to all chords during N2 show characteristic increases in the delta, theta, and alpha bands, followed by increases in the spindle and beta bands; (B) differences between the neural responses of major and minor chords in N2; (C) major and dissonant chords in N2; and (D) minor and dissonant chords in N2; (E) oscillatory responses to all chords presented in SWS. (F) Major and minor chords in SWS, (G) major and dissonant chords in SWS, and (H) minor and dissonant chords in SWS. We observed a generally weaker response in SWS in comparison to N2. Moreover, the response to major chords was consistently stronger than the response to the minor and dissonant chords (see text and Table 2 for significant clusters).

In addition to our ERP analysis, we conducted time-frequency analyses (TFAs). Musical chord presentation during NREM sleep elicited a well-known response pattern consistent with tone-induced K-complexes and spindle oscillations: increases in frequency power after several hundred of milliseconds in the SWA band (1–4.5 Hz), theta band (5–7 Hz) and alpha band (8–12 Hz), followed by increases in responses in the spindle band (slow spindles 11–13 Hz; fast spindles 13–15 Hz) and beta activity (15–25 Hz). The increases were generally more pronounced after chord presentations during N2 than in SWS (Figure 2, A and B).

Importantly, the increases differed according to the different musical chord types: Mean oscillatory response was consistently stronger after major chords than after minor or dissonant chords (see Figure 2). Particularly during N2 sleep, significant clusters occurred in the low frequency range (SWA and theta) as well as later higher-frequency ranges (alpha, spindles, and beta) when comparing major chords against the two other chord types. The increases in power were smaller and more specific in SWS sleep, encompassing mainly clusters in the higher frequency range (alpha, spindles, and beta). We did not observe a significant difference in the comparison of minor vs. dissonant chords. For a better overview, all significant clusters are presented in Table 3 and visualized in Figure 2.

**Table 3:** Significant TFA clusters of major, minor, and dissonant chords

			**Significant clusters**		
**Frontal** **frequency band**			Maj vs. Min	Maj vs. Dis	Min vs. Dis
	**Sleep Stage**	**Cluster**	Time in s	Time in s	Time in s
SWA (1–4.5 Hz)	N2	Cluster 1	0–2.60	0–2.60	No cluster
	SWS	No cluster			
Theta (5–7 Hz)	N2	Cluster 1	0.24–1.06	0.19–2.46	No cluster
		Cluster 2	1.18–1.70	2.67–3.18	
		Cluster 3	1.89–2.45		
	SWS	No cluster			
Alpha (8–12 Hz)	N2	Cluster 1	0.85–1.62	0.27–2.39	No cluster
		Cluster 2	2.65–3.06	2.70–3.36	
	SWS	Cluster 1	1.08–1.83	1.08–1.83	
		Cluster 2	0.50–0.89	0.50–0.89	
Slow spindles (11–13 Hz)	N2	Cluster 1	0.85–1.25	0.68–1.57	No cluster
	SWS	Cluster 1	0.94–1.96	0.94–1.96	
		Cluster 2	0.45–0.74		
Fast spindles (13–15)	N2	Cluster 1		0.79–1.27	No cluster
	SWS	Cluster 1	0.92–1.94	0.92–1.94	
		Cluster 2	2.48–3.02	2.48–3.02	
Beta (15–25 Hz)	N2	Cluster 1	0.80–1.17	0.76–1.59	No cluster
	SWS	Cluster 1	0.76–1.47	0.76–1.47	
		Cluster 2	2.51–3.01	2.51–3.01	

Significant clusters of time-frequency comparison between the different chord types in N2 and slow-wave sleep (SWS). SWA: slow-wave activity (1–4.5 Hz); Theta (5–7 Hz); Alpha (8–12 Hz); Slow spindles (11–13 Hz); Fast spindles (13–15 Hz); Beta (15–25 Hz). Clusters are given in seconds (s).

### Playing an instrument increased differentiation of musical chords during sleep

**Figure 3:**
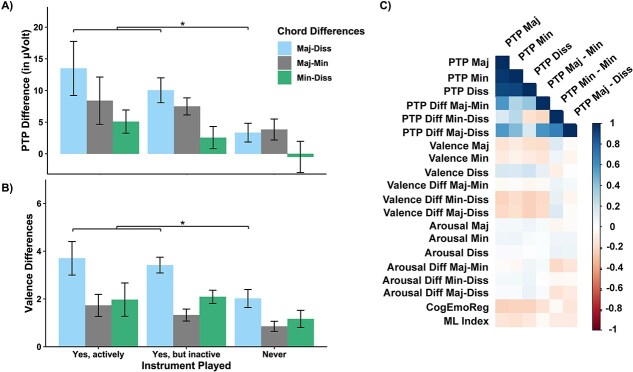
The effect of playing a musical instrument on PTP differences and differences in valence rating and correlation matrix. (A) PTP amplitudes (in μV) and (B) valence rating separated by chord differences Major-Dissonant, Major-Minor, and Minor-Dissonant for the variable “Instrument played” with the conditions “never,” “yes, actively,” and “yes, but inactive.” Values are means ± SEM. Participants who have played or still actively play an instrument showed a more pronounced ERP differentiation between Major and Dissonant chords compared to participants who never played an instrument. Similarly, valence ratings followed the same pattern, with significant differences indicated by ^*^: *p* < .05. (C) Pearson’s Correlation matrix visualizing the individual correlation coefficient between objective and subjective variables. PTP Maj: PTP Major; PTP Min: PTP Minor; PTP Diss: PTP Dissonant; PTP Diff Maj-Min: PTP Difference between major and minor chords; Valence Diff Maj-Min: Valence rating difference between major and minor chords; Arousal Diff Maj-Min: Arousal rating difference between major and minor chords. CogEmoReg: Cognitive Emotional Regulation through music; ML Index: Music listening index.

In subsequent analyses, we explored possible modulations of chord-induced brain responses during sleep by musical experience and subjective ratings, which have been observed during wakefulness, e.g. [18]. In particular, we observed that the differences in PTP amplitudes induced by musical chords presented during sleep depend on the ability to play a musical instrument (*F*(2,44) = 3.18, *p* = .051, *η _G_*  ^2^ = 0.13, see Figure 3, A): Participants who actively played an instrument (*n* = 9) had a PTP difference of 13.49 ± 4.27 μV between major and dissonant chords during sleep. Participants who did play an instrument but stopped (*n* = 12) had a difference of 10.04 ± 1.96 μV, while participants who never played any instrument had a PTP difference of 3.34 ± 1.48 μV. A Helmert contrast comparing the PTP differences of participants who play an instrument (active and inactive) vs. no instrument confirmed our observation (*t*(44) = −2.51, *p* = .016). We observed a similar pattern for the difference in PTP between major and minor chords processed during sleep, but no significance (*p* = .820). Actually, playing an instrument enabled the sleeping brain even to significantly differentiate between minor and dissonant chords during sleep when tested against zero (*t*(8) = 2.77, *p* = .024, *d* = 0.92). In contrast, we observed no such difference between these musical chord types in participants who used to play an instrument or not at all (*p* > .159).

As expected, playing an instrument also modulated subjective ratings during wakefulness of the different chord types, with higher differentiation with more musical experience (*F*(2,44) = 3.45, *p* = .041, *η _G_*  ^2^ = 0.14, see Figure 3, B). However, we did not observe any bivariate correlation between valence ratings of chord types during wakefulness and PTP amplitude or PTP amplitude differences during sleep. Similarly, no meaningful correlations occurred for arousal ratings, the emotional regulation score, or any other of the music expertise questionnaire (see Figure 3, C, for a summary).

## Discussion

In the current study, we presented isolated major, minor, and dissonant chords during NREM sleep stage N2 and SWS in a single night and investigated whether the sleeping brain is capable of processing musical harmony. We show that during both N2 and SWS, major chords consistently exhibited the largest PTP amplitude in the ERP during sleep compared to minor and dissonant chords. The differential response to musical chord types of the sleeping brain was largest for participants who played an instrument. Our results should be considered as proof-of-concept investigation providing clear, initial evidence that our brain is capable of processing harmonic information of music during sleep, and that this ability is modulated by musical experience.

The perception of musical harmony and dissonance depends both on the experience and cultural context of the listener and psychoacoustic features of the stimuli. The dissonant chord used in our study consisted of three tones separated by both one and two semitones. From a psychoacoustic perspective, these very small intervals of one or two semitones induce a low-level sensory perception of “dissonance” or “roughness” [10, 44]. It is worth noting that the differentiation between consonance and dissonance begins in very early processing stages of the auditory system, such as the primary auditory cortex (A1). Moreover, this differentiation has also been observed in several animal species and in studies on young infants without musical training, which even found a preference for consonance over dissonance [51–54]. Thus, it is likely that our reported differentiation of consonant (major and minor) vs. dissonant chord types during sleep is induced by low-level psychoacoustical processes and features.

However, this argument does not apply to the differentiation of major and minor chords, as they are both considered consonant. In addition, they both contain a three-semitone interval (minor third) and a four-semitone interval (major third). The only difference lies in the order of these two intervals: in a major chord, the major third builds the foundation, whereas in a minor chord, it appears between the two upper notes [3].

The valence evaluation of major vs. minor chords is strongly influenced by the cultural context [55]. For example, in cultures not familiar with Western music, major chords and melodies are not necessarily judged as “happier,” while this consistently occurs in cultures familiar with Western music [56, 57]. Thus, our reported differentiation of major and minor chords during sleep clearly shows that our brain processes chords also on the level of musical harmony beyond low-level acoustic features. Our finding that musical expertise modulates the brain responses to chord types during sleep corroborates this argument.

To our knowledge, this is the first experimental study examining the processing of musical harmony during sleep in humans. Our findings of differential brain responses to musical harmony are consistent with studies conducted during wakefulness, confirming distinct ERP responses [21–23], as well as differing brain activity patterns observed in brain imaging studies [9, 19]. Waking-EEG studies also reported influences of musical expertise [8, 18, 58, 59], concluding that non-musicians have less effective perceptual strategies for processing musical harmony and a different emotionally driven perception of chords compared to musicians.

Interestingly, while we did not observe different ERP responses during sleep between minor and dissonant chords, waking studies have reported differential responses to these two chord categories [9, 20]. One might argue that from a harmonic perspective, the dissonant chord used in our study has similarities to a minor chord, as it included a minor third between the root and highest note plus the ninth note of the minor scale. Still, during waking, participants strongly differentiated between dissonant and minor chords in valence and arousal. Furthermore, participants who played a musical instrument were actually capable of differentiating between minor and dissonant chords during sleep based on the ERP response.

Obviously, ERPs evoked during NREM sleep differ strongly in morphology and topography from those observed during wakefulness [60, 61]. The discrepancies between wake and sleep ERPs are attributed to the greater synchronous firing and inhibition of neurons during sleep, which result in higher ERP amplitudes compared to wakefulness [62]. Furthermore, sounds presented during NREM sleep can evoke K-complexes, which are characterized by very high negative amplitudes (<75 μV). Increasing slow waves by tones presented during NREM sleep is used to increase SWS in auditory closed-loop paradigms [30, 36, 63–65]. On average, sound presentation during NREM sleep typically elicits a positive peak (at approximately 200 ms) followed by a negative peak around 550 to 800 ms [50, 66]. Sound-induced negative peaks are typically higher and later in N2 sleep compared with SWS. The negative amplitude can vary in response to different stimuli [66], indicating a certain extent of stimulus processing during sleep [60]. For example, the negative amplitudes are generally elevated for deviant stimuli that differ in intensity and pitch when presented during NREM sleep [50].

Turning to the oscillatory response, we observed a very typical oscillatory response pattern induced by sound presentation during NREM sleep consistent with many other sleep studies (see e.g. [67],). Also, in the time-frequency domain, responses are typically higher during N2 sleep than SWS, consistent with the results reported here. In addition, oscillatory power in several frequency bands elicited by the major chords was significantly larger compared with power elicited by the minor and dissonant chords. These results also confirm that musical harmony is processed differently in the sleeping brain.

Limitations of our study are the use of only a small set of electrodes and a relatively low sampling rate. Thus, we are unable to identify brain regions that are involved in the processing of musical harmony during sleep given the poor spatial resolution of the EEG. Therefore, future studies should use high density EEG or used other brain imaging techniques like MEG or fMRI to provide more detailed information on where processing of musical harmony during sleep takes place in the brain [8, 18, 21, 22, 68]. In addition, given the dependency of musical harmony to the cultural context [55], it would be very relevant to test the ability of the sleeping brain to process musical harmony in different cultural contexts. Furthermore, it would be also relevant to use other chord types (e.g. Major 7 chords, inverted chords etc., see [13, 69]). to examine more closely the capacity of the sleeping brain to process musical harmony. Finally, as we have only used single chords in our study, future studies need to examine also harmonic sequences of chords to establish whether the sleeping can process musical harmony also in a certain musical context.

All in all, this is the first proof-of-concept study showing that the sleeping brain is capable to differentiate musical harmony during sleep, with musical expertise providing an advantage in distinguishing harmonic chord types. Moreover, this study paves the way for further research on music and harmony perception during sleep. Future research should provide, e.g. mechanistic insights on the contribution of subcortical brain areas. Moreover, future studies should examine musical expertise in more detail. In addition to musical training, individual musical preferences may also play an important role. This could be addressed in a new study by presenting short snippets of music from different genres. Furthermore, additional analyses of musical chord processing during wakefulness would also provide deeper insights into the particularities of ERP differences to musical chord processing during sleep. Finally, our findings that the sleeping brain is capable of processing harmonic information during sleep suggest that certain musical stimuli might also be capable of inducing specific emotions and mental states during sleep. Such stimuli could serve as cues similar to those used in TMR research. Given that music is inherently emotional and able to reactivate autobiographical memory [70, 71], it may be possible to use it not only to reinforce neutral memory traces associated with musical chords before sleep, but also to selectively reactivate emotional aspects of experiences during sleep. Future studies could use music to induce specific emotional states before sleep and examine whether exposure to different chord types during sleep elicits distinct emotional responses, with minor chords inducing sadness and major chords inducing happiness, and whether these responses affect emotional memory consolidation or dream content. Emotionality of dream content has already been successfully modulated by presenting odors during sleep [72]. Furthermore, presentation of relaxing words during sleep has been shown to extend time spent in SWS, decrease heart rate and improve subjective sleep quality [49, 73]. Thus, presenting exclusively major or minor chords during sleep or other relaxing musical stimuli may influence both objective and subjective sleep quality, but further studies are imperatively needed to clarify how musical harmony is perceived and processed during sleep. Overall, these current proof-of-concept results may lay the groundwork for developing interventions that use exposure to musical stimuli during sleep to reactivate memories and emotional mental concepts and possibly even improve sleep quality itself.

## Data Availability

Datasets analyzed for the current study are available online in Open Science Framework (OSF) at https://osf.io/9ywm2/.
